# A Fully Integrated Wireless System for Intracranial Direct Cortical Stimulation, Real-Time Electrocorticography Data Transmission, and Smart Cage for Wireless Battery Recharge

**DOI:** 10.3389/fneur.2014.00156

**Published:** 2014-08-25

**Authors:** Marco Piangerelli, Marco Ciavarro, Antonino Paris, Stefano Marchetti, Paolo Cristiani, Cosimo Puttilli, Napoleon Torres, Alim Louis Benabid, Pantaleo Romanelli

**Affiliations:** ^1^Computer Science Division, School of Science and Technology, University of Camerino, Camerino, Italy; ^2^Ab Medica Spa, Milan, Italy; ^3^Aethra Telecommunications Srl, Ancona, Italy; ^4^Clinatec, Laboratoire d’ Électronique des Technologies de l’Information (LETI), Commissariat à l’Energie Atomique et aux Energies Alternatives (CEA), Grenoble, France

**Keywords:** EcoG, epilepsy, seizures, RF, cortical stimulation, BCI

## Abstract

Wireless transmission of cortical signals is an essential step to improve the safety of epilepsy procedures requiring seizure focus localization and to provide chronic recording of brain activity for Brain Computer Interface (BCI) applications. Our group developed a fully implantable and externally rechargeable device, able to provide wireless electrocorticographic (ECoG) recording and cortical stimulation (CS). The first prototype of a wireless multi-channel very low power ECoG system was custom-designed to be implanted on non-human primates. The device, named ECOGIW-16E, is housed in a compact hermetically sealed Polyether ether ketone (PEEK) enclosure, allowing seamless battery recharge. ECOGIW-16E is recharged in a wireless fashion using a special cage designed to facilitate the recharge process in monkeys and developed in accordance with guidelines for accommodation of animals by Council of Europe (ETS123). The inductively recharging cage is made up of nylon and provides a thoroughly novel experimental setting on freely moving animals. The combination of wireless cable-free ECoG and external seamless battery recharge solves the problems and shortcomings caused by the presence of cables leaving the skull, providing a safer and easier way to monitor patients and to perform ECoG recording on primates. Data transmission exploits the newly available Medical Implant Communication Service band (MICS): 402–405 MHz. ECOGIW-16E was implanted over the left sensorimotor cortex of a macaca fascicularis to assess the feasibility of wireless ECoG monitoring and brain mapping through CS. With this device, we were able to record the everyday life ECoG signal from a monkey and to deliver focal brain stimulation with movement elicitation.

## Introduction

In patients with refractoryepilepsy or brain tumors, direct cortical electrical stimulation (DCS) and ECoG are the gold standard intraoperative technique to identify the tissue to be removed, particularly with regard to neighboring potentially eloquent cortex (1, 2). DCS requires the application of an electrical stimulus directly to the cortex to assess the contralateral muscle contraction in anesthetized patients. In addition, it can be used to generate transient behavioral effects in awake patients while they perform motor or cognitive tasks (3). DCS allows a precise mapping of the cortical organization of patients undergoing a resective procedure. Interindividual variability of cortical organization and changes of the location of function due to neural plasticity can be assessed by preoperative functional Magnetic Resonance Imaging (fMRI), but intraoperative DCS is needed to maximize the extent of the resection and to prevent neurological injury (4). ECoG has the additional opportunity to record the remote effect of electrocortical stimulation without distortion within a limited distance of a few millimeters, and can provide further details about the potential functional reorganization caused by the individuals brain pathology (3, 5). Moreover, given the high spatial (of ≈1–2 mm) and temporal resolution (within the timescale of neural activity), ECoG recording is required to identify an ictal focus and to guide the resection ECoG is also the most effective tool for brain–computer interface (BCI) applications. ECoG recordings from sensorimotor cortex are a promising tool to guide a prosthetic limb or an exoskeleton, thus providing functional restoration to patients with a variety of neurological injuries such as post-traumatic tetraplegia or paraplegia. In particular, the high signal-to-noise ratio of this technique enables the examination of high-frequency bands, unavailable for scalp EEG recordings, allowing to use the spectral analysis as a tool for brain functions mapping (6, 7) and BCI applications (8). The available commercial ECoG systems require cables leaving the skull of the patient to be connected with an external recording system, thus allowing only short term recordings. The cables connecting the electrodes placed on the cortex with the external apparatus leave the skull through a subcutaneous channel, which provides a path for infection and increases significantly the risk of bleeding and hemorrhage (9). Here, we described our preliminary experience with a chronically implantable wireless EcoG device. This device, custom-made for non-human primate use, provides wireless real-time EcoG recording and DCS. Here, we describe the device, the surgical protocol, and some preliminary results.

## Materials and Methods

### The fully implantable wireless system

**Figure 1:**
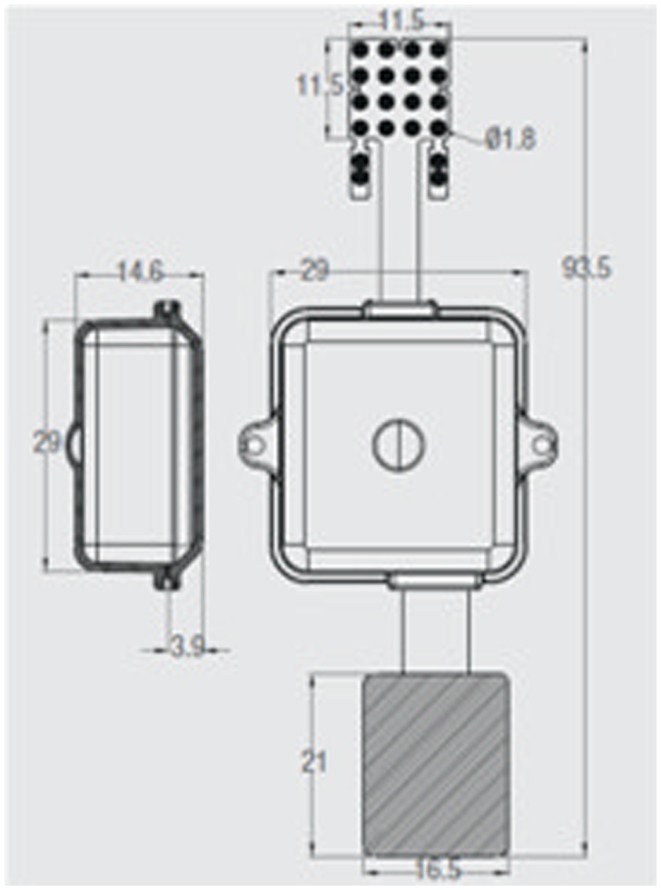
**System overview a fully implantable wireless system ECOGIW-16E**. The 16-electrodes grid, the PEEK body (lateral and axial views) and the RF antenna are visible.

**Figure 2:**
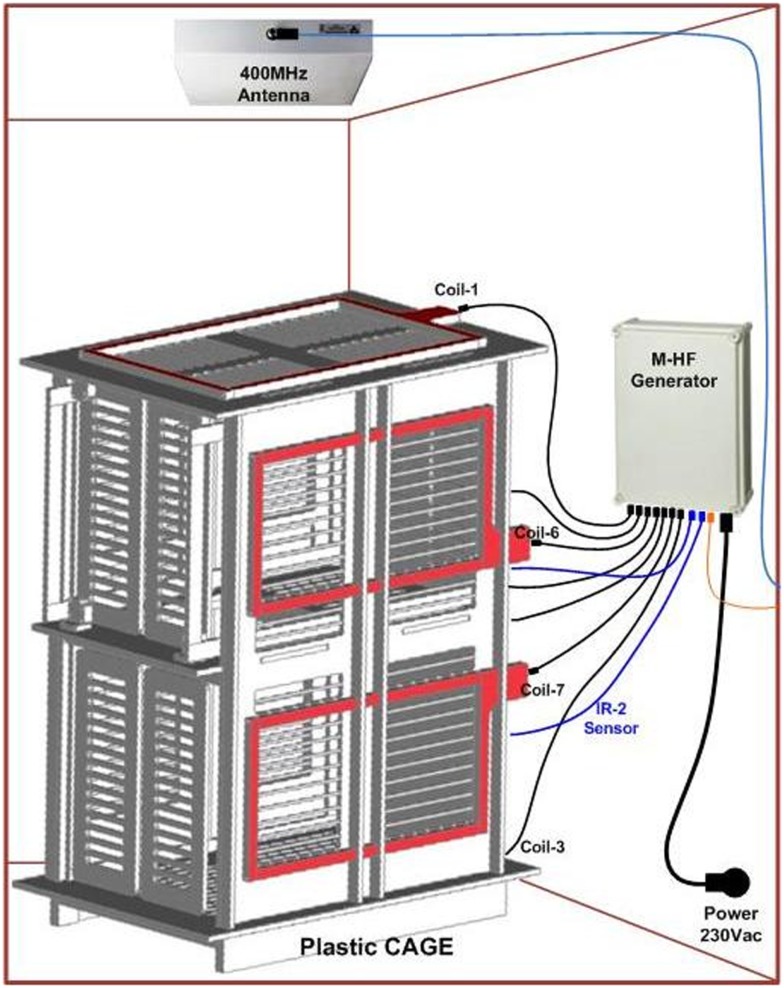
**System overviews a wireless recharge system for ECOGIW-16E**.

**Figure 3:**
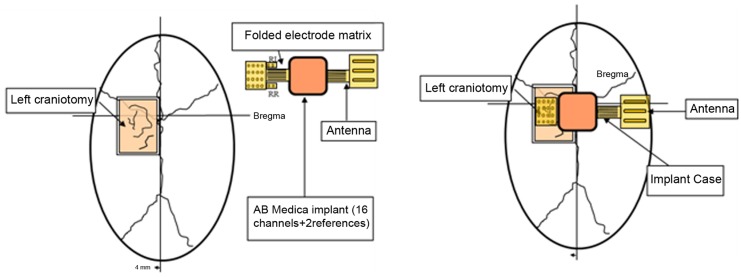
**Scheme showing the position of the device: it is made by three elements, the electrode grid, the antenna, and the body**. The body is in contact with the monkey skull and fixed to it using sutures and titanium screws. The electrode grid was in contact with the cortex and secure to the dura flap. In order to fit into the opening, we folded the portion connecting the grid and the case. The antenna of the device was in contact with the periosteal and the galea aponeurotica of the animal. All the system is covered by the animal skin.

ECOGIW-16E (Figure 1) was designed specifically to be tested in monkey. The total length of entire device is 9.35 cm and to achieve the best optimization and compactness, it consists of three parts: the electrode grid, the body, and the RF antenna. The grid is in contact with the cortex and secured to the dura flap; it is made by a single sheet of flexible polyimide support that integrates 16 electrodes with a spacing of 7.6 mm and an exposed surface of 1.8 mm in diameter, covered by a 300 μm layer of platinum; the body part made by PEEK include a microcontroller that handles local processing and the transceiver module for implantable medical applications within MICS band with a 800/400/200 kbps raw data rate. The microprocessor used was ARM^®^CortexTM-M4 MCUs as the Kinetis MK40N512VMD100 from Freescale. We used also an analog front-end by Texas Instruments, the ADS1298. It is a multichannel, simultaneous sampling with 16-bit, delta-sigma (ΔΣ) analog-to-digital converters (ADCs), and a built-in programmable gain amplifier (PGA). We recorded ECoG signals from implanted grids using a sampling rate of 512 Hz (@16 bit), in any way our device has a programmable sampling rate from 250 Hz to 2 KHz. In addition, the body includes a triaxial accelerometer, a stimulus generator, a sensor of temperature/load current, and a Li-Ion battery (3.6 V 350 mA/h ISO 13485). It is in contact with the monkey skull and tightly fixed to it using sutures and titanium screws. In order to fit into the opening, we folded the portion connecting the grid and the case (Figure 3). The antenna of the device was in contact with the periosteal and the galea aponeurotica of the animal. All the system is covered by the animal skin. The entire device is covered with a parylene coating of 7 m to ensure maximum biocompatibility. Finally, we have adopted a charging apparatus to provide an induction charger (250 mW, 70 mA @ 3.7 V) for charging a wireless rechargeable battery. The interface consumes 58 mA (16CH @ 500SPS + TX-RF), 30 mA (16CH @ 500SPS), and 7 mA in standby. The link module for wireless connection is Zarlink. ECOGIW-16E is wirelessly inductively rechargeable (at 150 kHz) using a special designed cage for recharge, thus allowing to overtake the problems of the current ECoG systems due to the cable connection and the necessity of sedation period for recharging. Using the smart wireless recharge cage, it is also possible to recharge the implanted device during the physiological rest period. In Figure 2, it reported the complete recharging cage system designed and produced by Aethra Communication company (Ancona, Italy).

### Surgical procedure

One male macaque monkey (*Macaca fascicularis*), weighing 6.95 kg, was used in this study. Experimental protocol was approved by the regional committee (Cometh Grenoble) and registered to the national committee under the number 12/136 Clinatec-NTM-01 and complied with the EU directive 22nd September 2010 (2010/63/EU) on the care and use of laboratory animals. MRI was performed prior to surgical exploration to define surgical management. The animal was anesthetized using Xylazine (5 mg/kg), and Ketamine hydrochloride (20 mg/kg), intramuscular (IM), and then a maintenance dose of 1.25 mg/kg, 5 mg/kg Xylazine/Ketamine. Heart rate, blood pressure, respiratory depth, and body temperature were constantly monitored by veterinary staff. Surgical procedures took place in standard aseptic conditions. When deep anesthesia was achieved, the animal was secured to a stereotaxic frame, and a craniotomy was performed over the left motor cortex (M1) in Brodmann area 4. First, a rectangular (2.5 cm × 2 cm) bone portion was removed, then the dura mater was cut in Y shape, and the leaves were retracted to expose the surface of the M1. The location of the grid electrodes was determined by identification of anatomical landmarks such as the central sulcus, the intraparietal sulcus, and the arcuate sulcus. Moreover, radiographic images during surgery were acquired to guide device placement. The device was positioned orthogonally and the grid was centered above the hand knob of the left motor cortex (Figure 5).

## Results and Discussion

**Figure 4:**
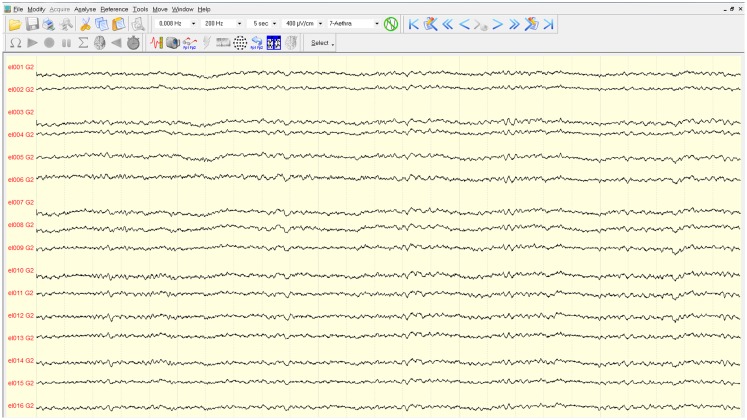
**Traces of the EcoG**. Signals are filtered with a pass-band filter from 0.008 to 200 Hz. The time interval between two vertical gray lines is 5 s.

The mapping procedure was performed basically in two steps. In the first one, we record the EcoG signal of the monkey obtaining some traces like the ones in Figure 4; the signals are recorded using a commercial software for EcoG recording provided by mycromed, Treviso, Italy and the signal was recorded using a 512 Hz sampling rate, then the impedence in each electrode was constantly monitored too (Figure 6). In the second step, we performed bipolar stimulation by pulses of rectangular shapes with anodal monophasic current pulses of 0.5 ms duration. This stimulation technique consists of a train of five pulses delivered at 1 Hz with an interstimulus interval of 100 ms.

**Figure 5:**
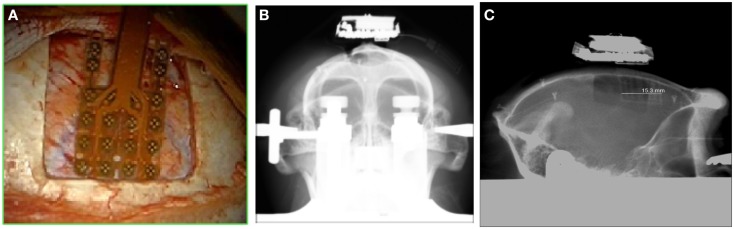
**Location of the grid on the cortex (A); frontal X-ray showing the position of the whole device (B) and the lateral view (C)**.

**Figure 6:**
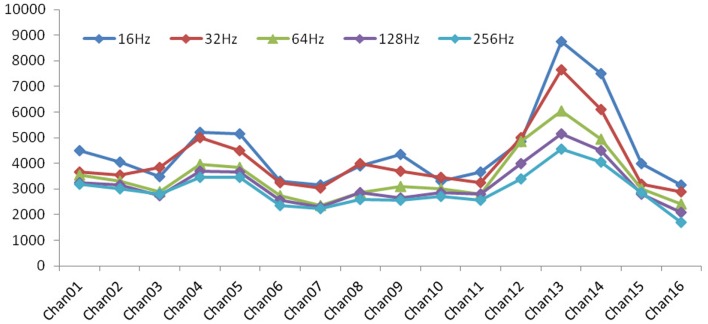
**Impedence in each electrode: with increasing of frequency, the values of impedance exhibited a general decrease, which is a characteristic behavior for a general electrode–electrolyte interface**.

**Figure 7:**
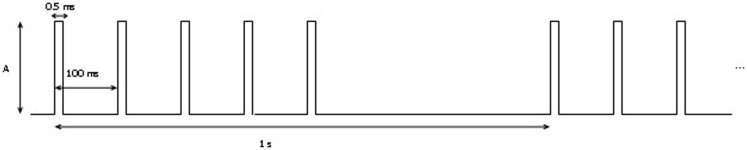
**Stimulation protocol: A represents the amplitude of the signal**. It was increased from 1 to 3 mA by 0.5 A per step.

Stimulus intensity was gradually increased in increments of 0.5 mA, starting at 1 mA up to a maximum of 3 mA (Figure 7). We test all the contact with a reference electrode positioned in the left of grid while the ones on the right were off. During cortical stimulation of the expected motor cortex, movements of distinct portions of the right arm were observed with a stimulation intensity of 2 mA. Specifically, we first demonstrate that the stimulation with electrode number seven and nine elicit movements of the proximal portion of the right arm, whereas the stimulation with electrodes number one generates movements of the distal portion of the right arm.

In conclusion, our results suggested that technology that incorporates an MICS interface and a microcontroller handling local processing can be used to generate stimuli for intraoperative functional brain mapping and transmit the ECoG signals in real time. Furthermore, these observations suggest that this device can be used to map the seizure focus during epilepsy procedures and for clinical application of invasive BCI. The advantages of fully implantable integrated system derive from the absence of connecting cables, with improved safety and patients comfort. This fully integrated system lends itself to be optimized as closed-loop system of electrical stimulation for aborting or blocking promptly detected seizure activity in epilepsy patients. Further experimental activity is underway to study the long-term tolerability of the system on primates.

## Conflict of Interest Statement

The authors declare that the research was conducted in the absence of any commercial or financial relationships that could be construed as a potential conflict of interest.
